# A Bibliometric Analysis of Publications on Oxycodone from 1998 to 2017

**DOI:** 10.1155/2019/9096201

**Published:** 2019-10-31

**Authors:** Fan Lei, Jishi Ye, Juan Wang, Zhongyuan Xia

**Affiliations:** Department of Anesthesiology, Renmin Hospital of Wuhan University, Wuhan 430060, Hubei, China

## Abstract

**Background:**

Oxycodone is a widely used opioid analgesic, which is involved in cancer pain and non-cancer pain. This study is intended to understand the publication characteristics of oxycodone research field and assess the quality of pertinent articles from 1998 to 2017.

**Methods:**

Oxycodone-related publications from 1998 to 2017 were retrieved from the Web of Science (WOS) and PubMed database. These papers were coded across several categories, such as total number, journals, countries, institutions, authors and citations reports. And the analysis of co-occurrence keywords was handled by VOSviewer software.

**Results:**

According to search strategies, a total of 2659 articles on oxycodone were published in world from 1998 to 2017 in WOS. Among the top 10 most productive organizations, six of them were American institutes, two of them were pharmaceutical enterprises and the other three were Finnish, Australian and Canadian institutes, which is similar with the distribution by country/region. Drewes AM from Denmark published most articles and PAIN MEDICINE is the most productive journal in oxycodone area. Meanwhile, clinical studies occupy a dominant position during the past 20 years. The 10 most cited papers were listed. Among these articles, 8 of them are reviews and 2 of those are meta-analysis. And the last decade (2008–2017) displayed that the newest keywords focus on “double-blind”, “randomized controlled trial” and “neuropathic pain”.

**Conclusions:**

The findings provided a comprehensive overview of oxycodone research. In view of the adverse effects of oxycodone, high-quality oxycodone studies both in basic studies and clinical trials need to be completed.

## 1. Introduction

In the last 20 years, great advances had been made in the field of pain research. At the same time, different pain-related drugs have also continued to emerge. Generally speaking, pain relieving drug could be classified into two parts: opioids and nonopioids [1–3]. Among many opioid agents, oxycodone occupied an important position. In 1917, oxycodone was first synthesized from thebaine in Germany laboratory. As a selective mu-opioid receptor agonist with potential kappa-opioid receptor agonism, oxycodone was broadly used in all kinds of field, such as postoperative pain, neuropathic pain and cancer pain [4, 5]. Pain study, especially for analgesic, is a robust research area, which attracted much attention and obtained numerous supports from governments and pharmaceutical companies. As a result, many studies, especially for oxycodone, became more and more, no matter involving animal research or clinical trials. However, the current state of oxycodone research and the dynamic change of hotspots have not been detected and analyzed.

Bibliometrics is statistical analysis of written publications and now commonly used to analyze research topics, research status, and publication quality by a series of quantitative tools [6]. Bibliometric methods are frequently used in many research areas, including critical care [7], diabetes [8, 9], low back pain [6] and cancer [10]. Using these methods, we could trace the relationship amongst academic journal citations and make sure the trends of research directions to provide an overview of oxycodone research and formulate the future development strategy. With rapid growth of oxycodone-related publications, it is necessary to comb through and classify the massive publication from different aspects, including countries/region, journals, authors, categories, institutes, keywords and citations.

To analyze the research trends and summarize the characteristics of publications regarding oxycodone within the past 20 years, we performed a bibliometric analysis of related references derived from the Web of Science (WOS) database and PubMed database by built-in tool of WOS and VOSviewer software.

## 2. Methods

### 2.1. Bibliometric Data

This study was conducted based on methodology of previous similar articles. All data were acquired on October 31, 2018. Considering that these data were downloaded from public databases and there existed no ethical questions about them, we did not apply for ethical approval. oxycodone-related papers published between 1998 and 2017 were retrieved from PubMed database and the Web of Science (WOS) online database, which included the Science Citation Index Expanded (SCIE), Social Sciences Citation Index (SSCIE) and Arts & Humanities Citation Index(A&HCI). The journal impact factors (IF) came from Journal Citation Reports 2017 database. In addition, research types, including basic research, randomized controlled trials (RCTs), clinical trials, and case reports, were retrieved from the PubMed database.

### 2.2. Search Strategy

In WOS, the search terms were as follows: TOPIC: (oxycodone) Refined by: DOCUMENT TYPES: (ARTICLE OR PROCEEDINGS PAPER OR REVIEW OR MEETING ABSTRACT) AND LANGUAGES: (ENGLISH) Indexes = SCI-EXPANDED, SSCI, A&HCI, CPCI-S, CPCI-SSH, ESCI, CCR-EXPANDED, IC Timespan = 1998–2017. In PubMed, the search terms were as follows: "oxycodone"[MeSH Terms] OR "oxycodone"[All Fields]) AND (("1998/01/01"[PDAT]: "2017/12/31"[PDAT]) AND English[lang]. Literature type included basic research, review, randomized controlled trials, clinical studies and case reports. To search for basic research, we identified the species as “other Animals.” Two authors independently did the data extraction. The title and the abstract of potentially eligible articles were reviewed. Articles unrelated to oxycodone themes were further excluded.

### 2.3. Bibliometric Analysis

Descriptive statistics are mainly used in this study. Research trends and Publication features, including the distribution of countries/regions, institutions, total citations, average citations per item, h-index, journals, authors and research areas were classified and analyzed by the intrinsic function of Web of Science. Besides, the top 10 most cited articles were also listed. In addition, using VOSviewer (Van Eck & Waltman, Leiden University, The Netherlands) and Microsoft Excel, we performed data mining and mapping and clustering of the retrieved publications.

## 3. Results

### 3.1. Oxycodone-Related Publications Overview

**Figure 1:**
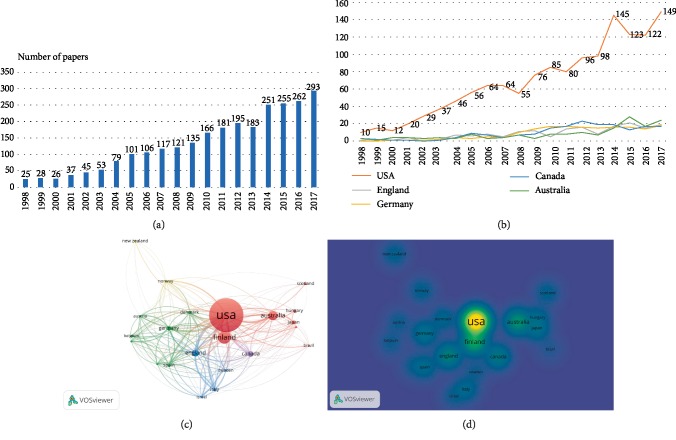
Oxycodone-related articles overview. (a) The global number of oxycodone publications. The blue bars indicate the quantity of oxycodone articles. (b) The time curve of oxycodone-related articles from top 5 countries. (c) The network map showing the relations between various countries in oxycodone research field. (d) The density map showing the intensity of various countries in oxycodone research field.

According to search strategies, a total of 2659 and 2702 articles on oxycodone were published in world from 1998 to 2017 in WOS and PubMed database, respectively. In WOS database, the number of oxycodone-related publications exhibited a positive trend, from 25 in 1998 to 293 in 2017. The United States ranked first, which published 1382 oxycodone-related articles (51.974%), followed by Canada (187, 7.033%) and England (174, 6.544%). Nearly 42 countries and regions involved in oxycodone research. Unlike the publication trend of USA, the annual publications of oxycodone in other developed countries have shown no substantial increase (Figures 1(a) and 1(b)). There is a considerable gap on quantity of literature between USA and other main countries.

In the network map of VOSviewer analysis, USA was in the central position of oxycodone research and closely coordinated with many countries, such as Finland, Australia and Japan.

Beyond that, multiple research collaborations also could be found between Germany and other countries, such as Danmark, Austria, and Belgium, which may be another oxycodone research center (Figures 1(c) and 1(d)).

### 3.2. Most Productive Organizations and Their Collaborations

**Figure 2:**
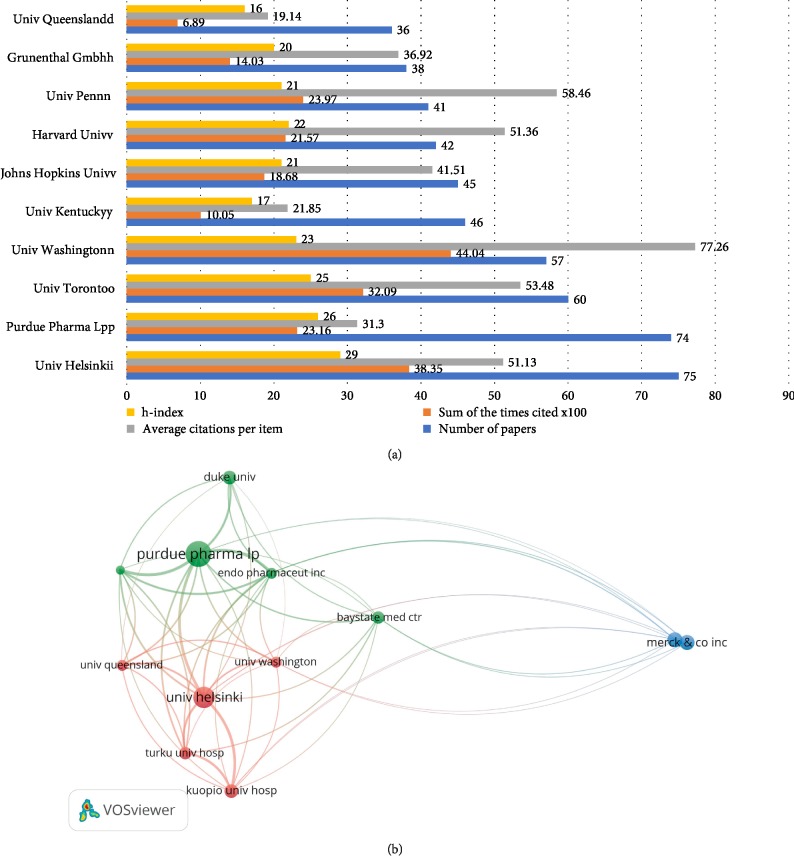
Most productive organizations and their collaborations. (a) Most productive organizations and their citation reports. (b) The network map of VOSviewer analysis showing the collaborations between various organizations in oxycodone area.

The top 10 most productive organizations are listed in Figure 2. UNIV HELSINKI in Finland contributed the most literatures with 75 oxycodone-related papers published, followed by PURDUE PHARMA (75 papers), and UNIV TORONTO (60 papers). Among the top 10 most productive organizations, six of them were American institutes, two of them were pharmaceutical enterprises and the other three were Finland, Australian and Canada institutes, which is similar to the distribution by country/region.

For publication quality, UNIV WASHINGTON had the first place, which had 4404 total citations, 77.27 average citations per item and 23 h-index. There is no obvious gap on quality of articles between USA institutions and other countries' institutions.

In the VOSviewer analysis, the relationships between University Helsinki with its collaboration were visualized. Visualization map of organizations grouped into 2 clusters. Except for the above cluster, Purdue Pharma also cooperated with many other pharmaceutical enterprises, such as Merck company and Endo pharmaceutical company.

### 3.3. Distribution by Author

**Figure 3:**
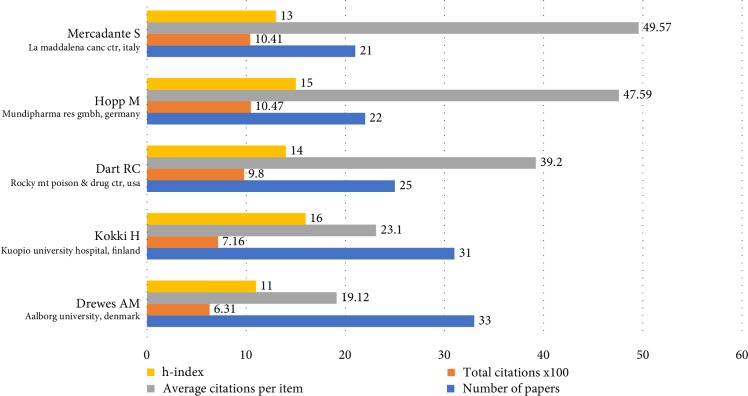
Distribution by authors. The top 5 active authors and their citation reports in oxycodone area are listed.

A total of 8867 authors participated in oxycodone related documents. As shown in Figure 3, the top 5 active authors and their citations report were listed. Drewes AM from Aalborg University, Denmark published most oxycodone-related articles, followed by Kokki H (Kuopio University Hospital, Finland) and Dart RC (Rocky Mt Poison & Drug Ctr, Denver, CO USA). And Hopp M ranked first in total citations, which was from Mundipharma Res GmbH & Co KG (Germany).

### 3.4. Distribution of Journals Publishing Oxycodone

**Figure 4:**
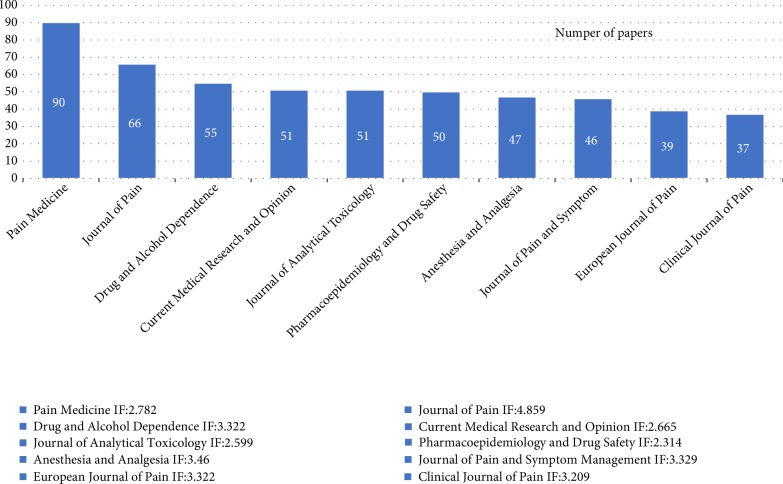
Distribution of journals publishing oxycodone. The main journals publishing oxycodone and their impact factors are listed.

532 articles regarding oxycodone published in the top 10 journals (20.008% of all literatures; Figure 4). PAIN MEDICINE produced the most papers (90, 3.385%), followed by JOURNAL OF PAIN (66, 2.482%) and DRUG AND ALCOHOL DEPENDENCE (55, 2.068%). According to Journal Citation Reports (2017 version), of the top 10 journals, PAIN MEDICINE, JOURNAL OF PAIN, DRUG AND ALCOHOL DEPENDENCE, CURRENT MEDICAL RESEARCH AND OPINION, ANESTHESIA AND ANALGESIA and JOURNAL OF PAIN AND SYMPTOM MANAGEMENT were classified in Q1. Other four journals were classified in Q2. The top 10 most productive journals and their impact factor (2017 version) were listed in Figure 4.

### 3.5. Research Types and Categories in the Oxycodone Field

**Figure 5:**
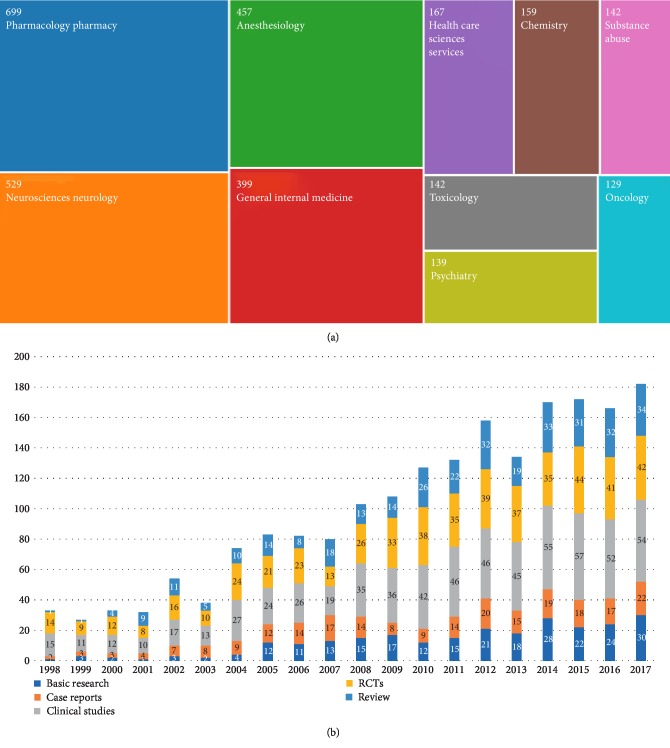
Research types and categories in the oxycodone field. (a) The research categories on oxycodone in world. (b) The article types analysis regarding oxycodone in the past two decades worldwide.

Based on the results of WOS and PubMed database, research types and categories in oxycodone field were listed in Figure 5. In the oxycodone research field, clinical studies has occupied a dominant position for the past 20 years. And the number of basic research papers also increased year after year. Meanwhile, the number of other research types maintained steady growth.

A total of 49 research categories involved oxycodone-related articles, globally. Pharmacology pharmacy is the main research category, with 669 papers (26.288%), followed by neurosciences neurology (529, 19.895%) and anesthesiology (457, 17.187%).

### 3.6. The Top 10 Most Cited Articles regarding on Oxycodone

As shown in Table 1, we listed the top10 most cited articles in the oxycodone field. The top10 articles on oxycodone research mainly concentrated in the period from 1999 to 2009. Among these articles, 8 of them are reviews and 2 of those are systematic review or meta-analysis. The most cited article is a review on chronic opioid therapy in chronic noncancer pain, which published in JOURNAL OF PAIN by Chou, Roger et al. “Opioid therapy for chronic pain” by Ballantyne, et al. was published in NEW ENGLAND JOURNAL OF MEDICINE, which is the top journal worldwide with IF 79.26.

### 3.7. Analysis of Keywords in Oxycodone-Related Publications

**Figure 6:**
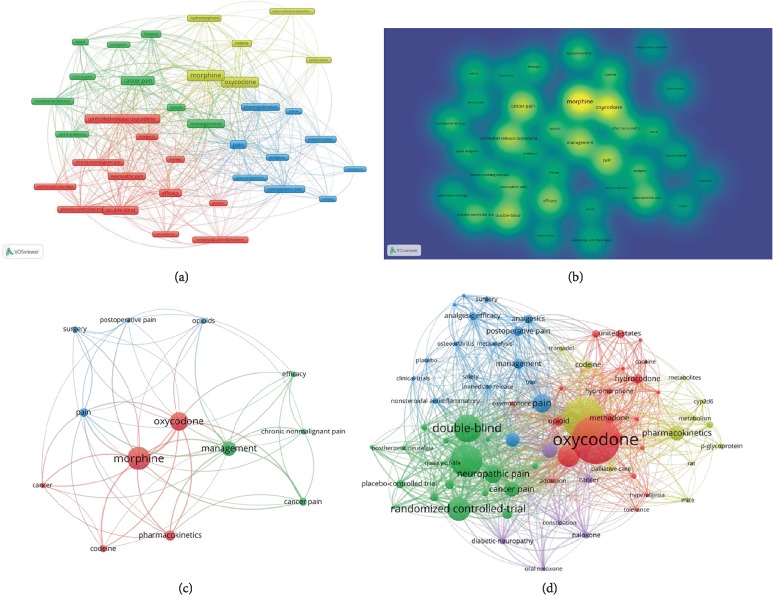
Analysis of keywords in oxycodone-related publications. (a) The analysis of co-occurrence keywords during two decades. (b) The density map of keywords in the past 20 years. (c) The analysis of co-occurrence keywords between 1998 and 2007. (d) The analysis of co-occurrence keywords between 2008 and 2017.

To detect directions and topics in oxycodone research field and understand the discipline development, we analyzed the distribution of co-occurrence keywords (minimum number of occurrences of a keyword is 10 times in titles and abstracts in all publications) by using VOSviewer software. In Figure 6, 36 identified keywords met the threshold were classified into the 4 clusters: “drugs”, “postsurgical pain”, “cancer pain” and “neuropathic pain” (Figure 6(a)). In the “drugs” cluster, the most popular keywords were: “morphine”, “oxycodone” and “codeine”. Other three clusters are main indications.

Compared with the period from 1998 to 2007, we discovered dynamic change of research popular topics with time. (5 times was set the minimum number of occurrences of a keyword in titles and abstracts in all publications). Overall, with the development of oxycodone research, co-occurrence keywords became more and more. In the early stage of oxycodone research (1998–2007), the main popular topics are little. The last decade (2008–2017) displayed that the newest keywords focus on clinical research and treatment, such as “double-blind”, “randomized controlled trial” and “neuropathic pain” (Figures 6(c) and 6(d)).

## 4. Discussion

Our bibliometric analysis presented a comprehensive overview of the trend and development of the scientific output in the oxycodone field over two decades. In general, the number of oxycodone-related articles increased nearly tenfold since 1998. No matter in data representation or VOSviewer analysis, the USA still held a pivotal position of oxycodone field. Nevertheless, compared to other fields, such as anesthesiology, respiratory and nanomedicine research, the number of papers and the degree of emphasis on oxycodone research are far low. According to some literatures, use of opioid analgesics has increased, but remains low in Africa, Asia, Central America, the Caribbean, South America, and eastern and Southeastern Europe, which also explained why most of oxycodone-related papers were from north America and western countries [3, 11]. Identified barriers to access to opioid analgesics urgently need to be addressed by governments and international agencies and so does the oxycodone research.

Similar with the distribution by countries and authors, the most productive organizations are mostly from the USA. It is noteworthy that pharmaceutical enterprises play an important role in the oxycodone research area. To increase the drug sale and gain the trust of doctors, pharmaceutical companies had to perform clinical trials with related institutes and hospitals to verify the safety and effectiveness of oxycodone, which is a vital force in global drug markets. Among the journals publishing oxycodone, most of them were classified into Q1 according to 2017 version Journal Citation Reports, suggesting that these studies also have high research value and could be widely spread. However, most of the journals publishing oxycodone almost focused on pain research, especially on clinical studies, which is largely consistent with the research types of oxycodone. Unlike other anti-tumor drug, the existing literatures of oxycodone mainly concentrated on clinical studies or RCTs. With the increase of clinical trials and evidence, the indications of oxycodone also expanded from cancer pain to non-cancer pain, including neuropathic pain, chronic pain and post-surgical pain. However, the research on the related mechanism of analgesia or biological signaling pathway are few. Actually, several basic studies also provided some novel perspectives for protective effects or addiction of oxycodone in animal models. Walentiny et.al found that nociceptin/orphanin FQ (NOP) receptor activation can modulate the discriminative stimulus effects of oxycodone in C57BL/6 mice [12]. In vivo and vitro, some results of researches also demonstrated that oxycodone could decrease spinal nerve injury (SNL)-induced activation of glial cells (astrocytes and microglia) and plasma levels of proinflammatory cytokines (IL-6, IL-1β and TNF-α) [13, 14]. In consideration of opioids-drugs abuse, Zhang et al. found several alterations of expression of genes related to inflammation/immune functions in the dorsal striatum of adult C57BL/6J mice following chronic oxycodone self-administration, which revealed some novel neurobiological underlying mechanisms of abused prescription opioid [15].

In the list of the top10 most cited articles regarding on oxycodone, 8 of them are reviews and 2 are systematic review or meta-analysis. These articles all involved in the following topics: opioid tolerance, opioid-induced abnormal pain sensitivity and immune modulation. It's conceivable that in the future, these directions are still the hot areas of oxycodone or opioid drugs research. Exploration of the change of keywords in the past two decades is helpful to grasp the development pulse of oxycodone studies. Over the past 20 years, 36 keywords were identified and classified into the 4 clusters: “drugs”, “postsurgical pain”, “cancer pain” and “neuropathic pain”, which reflected the main indications of oxycodone or opioid drugs. And compared to the period during 1998–2017, the last decade (2008–2017) appeared more co-occurrence keywords and the newest keywords focus on clinical research and treatment, such as “double-blind”, “randomized controlled trial” and “neuropathic pain”. It is well known that RCT is the highest level of evidence in evidence-based medicine. To verify the indications of oxycodone, many randomized controlled trials on oxycodone had been performed. So, it is not surprising that “randomized controlled trial” and “double blind” as keywords appeared more times in the recent 10 years. Although opioid drugs, including oxycodone, are widely used to treat neuropathic pain, and are considered effective by some professionals, only a few of low quality evidence showed that oxycodone is of value in the treatment of diabetic neuropathic pain or post-herpetic neuralgia. Maybe oxycodone or opioid drugs cannot do everything.

Some limitations still existed in our studies. Firstly, this analysis only included English articles. Therefore, there is some bias in current publications. Secondly, although WOS and PubMed database had the most complete coverage of articles, some papers from different database cannot be retrieved, which might lead to incomplete data collection and different results. Thirdly, for practical reasons, only the abstract, not the full text, was imported into VOSviewer software to analyze the co-occurrence keywords. These operations could not provide sufficient details on oxycodone research within the abstract. In addition, since the citations contained self-citations, we did not account for self-citations in our analysis, which may contain some controversial literature. Lastly, some articles included by related database may be delayed so the citations and h-index have existing flaws.

## 5. Conclusion

Despite these limitations, this extensive bibliometric analysis provides a comprehensive overview of the hotspots within oxycodone-related publications over 20 years. The findings illustrate a considerable growth in the number of papers of oxycodone research. Content and quality analysis revealed American institutes occupied a central position in the oxycodone research area. Meanwhile, pharmaceutical companies also played an enormous role in the promotion of oxycodone. In view of the addiction and adverse effects of oxycodone and opioid drugs, there are a lot of things to do, various countries should increase support to complete high-quality oxycodone studies both in basic studies and clinical trials.

## Figures and Tables

**Table 1:** The top 10 most cited articles on oxycodone research.

Title	First authors	Journals	Year	Total citations	Impact factor	Quartile in category
Clinical guidelines for the use of chronic opioid therapy in chronic noncancer pain	Chou, Roger	Journal of Pain	2009	1150	4.859	Q1
Pharmacologic management of neuropathic pain: Evidence-based recommendations	Dworkin, Robert H.	Pain	2007	1064	5.559	Q1
Efficacy of pharmacological treatments of neuropathic pain: an update and effect related to mechanism of drug action	Sindrup, S. H.	Pain	1999	773	5.559	Q1
Algorithm for neuropathic pain treatment: An evidence based proposal	Finnerup, N. B.	Pain	2005	726	5.559	Q1
Opioids in chronic non-cancer pain: Systematic review of efficacy and safety	Kalso, E.	Pain	2004	670	5.559	Q1
Opioid complications and side effects	Benyamin, Ramsin	Pain Physician	2008	592	2.556	Q3
Opioid therapy for chronic pain	Ballantyne, J. C.	New England Journal of Medicine	2003	551	79.26	Q1
Morphine and alternative opioids in cancer pain: The EAPC recommendations	Hanks, G. W.	British Journal of Cancer	2001	493	5.992	Q1
EFNS guidelines on pharmacological treatment of neuropathic pain	Attal, N.	European Journal of Neurology	2006	490	4.621	Q1
Opioids for chronic noncancer pain: A meta-analysis of effectiveness and side effects	Furlan, Andrea D.	Canadian Medical Association Journal	2006	464	6.818	Q1

## Data Availability

The datasets analyzed during the current study are available from the corresponding author on reasonable request.

## References

[1] Caes L., Boerner K. E., Chambers C. T. (2016). A comprehensive categorical and bibliometric analysis of published research articles on pediatric pain from 1975 to 2010. *Pain*.

[2] Riado M. D., Kowalski M., Vallve O. M. (2017). Methodological and reporting quality of systematic reviews published in the highest ranking journals in the field of pain. *Anesthesia & Analgesia*.

[3] Robert C., Wilson C. S., Lipton R. B., Arreto C. D. (2017). Growth of headache research: a 1983–2014 bibliometric study. *Cephalalgia*.

[4] Gkegkes I. D., Minis E. E., Iavazzo C. (2018). Oxycodone/naloxone in postoperative pain management of surgical patients. *Journal of Opioid Management*.

[5] Wirz S., Ellerkmann R. K., Soehle M., Wirtz C. D. (2018). Oxycodone is safe and effective for general anesthesia. *Journal of Opioid Management*.

[6] Liang Y. D., Li Y., Zhao J., Wang X. Y., Zhu H. Z., Chen X. H. (2017). Study of acupuncture for low back pain in recent 20 years: a bibliometric analysis via CiteSpace. *Journal of Pain Research*.

[7] Li Z., Liao Z., Wu F. X., Yang L. Q., Sun Y. M., Yu W. F. (2010). Scientific publications in critical care medicine journals from Chinese authors: a 10-year survey of the literature. *The Journal of Trauma: Injury, Infection, and Critical Care*.

[8] Gao Y., Wang Y., Zhai X. (2017). Publication trends of research on diabetes mellitus and T cells (1997–2016): a 20-year bibliometric study. *PLoS One*.

[9] Sweileh W. M. (2018). Analysis of global research output on diabetes depression and suicide. *Annals of General Psychiatry*.

[10] Zhu X., Zhou X., Zhang Y., Sun X., Liu H., Zhang Y. (2017). Reporting and methodological quality of survival analysis in articles published in Chinese oncology journals. *Medicine (Baltimore)*.

[11] Bosetti C., Santucci C., Radrezza S., Erthal J., Berterame S., Corli O. (2019). Trends in the consumption of opioids for the treatment of severe pain in Europe, 1990–2016. *European Journal of Pain*.

[12] Walentiny D. M., Wiebelhaus J. M., Beardsley P. M. (2018). Nociceptin/orphanin FQ receptors modulate the discriminative stimulus effects of oxycodone in C57BL/6 mice. *Drug and Alcohol Dependence*.

[13] Yang P. P., Yeh G. C., Huang E. Y., Law P. Y., Loh H. H., Tao P. L. (2015). Effects of dextromethorphan and oxycodone on treatment of neuropathic pain in mice. *Journal of Biomedical Science*.

[14] Ye J., Yan H., Xia Z. (2018). Oxycodone ameliorates the inflammatory response induced by lipopolysaccharide in primary microglia. *Journal of Pain Research*.

[15] Zhang Y., Liang Y., Levran O. (2017). Alterations of expression of inflammation/immune-related genes in the dorsal and ventral striatum of adult C57BL/6J mice following chronic oxycodone self-administration: a RNA sequencing study. *Psychopharmacology (Berl)*.

